# The rs13388259 Intergenic Polymorphism in the Genomic Context of the *BCYRN1* Gene Is Associated with Parkinson's Disease in the Hungarian Population

**DOI:** 10.1155/2018/9351598

**Published:** 2018-04-03

**Authors:** Sándor Márki, Anikó Göblös, Eszter Szlávicz, Nóra Török, Péter Balicza, Benjamin Bereznai, Annamária Takáts, József Engelhardt, Péter Klivényi, László Vécsei, Mária Judit Molnár, Nikoletta Nagy, Márta Széll

**Affiliations:** ^1^Department of Medical Genetics, University of Szeged, Somogyi u. 4, 6720 Szeged, Hungary; ^2^Department of Dermatology and Allergology, University of Szeged, Korányi fasor 6, 6720 Szeged, Hungary; ^3^MTA-SZTE Dermatological Research Group, University of Szeged, Korányi fasor 6, 6720 Szeged, Hungary; ^4^Department of Neurology, University of Szeged, Semmelweis u. 6, 6725 Szeged, Hungary; ^5^Institute of Genomic Medicine and Rare Disorders, Semmelweis University, Tömő u. 25-29, 1083 Budapest, Hungary; ^6^Department of Neurology, Semmelweis University, VIII. Balassa J. u. 6, 1083 Budapest, Hungary; ^7^MTA-SZTE Neuroscience Research Group, University of Szeged, Semmelweis u. 6, 6725 Szeged, Hungary

## Abstract

Parkinson's disease (PD) is a common neurodegenerative disorder characterized by bradykinesia, resting tremor, and muscle rigidity. To date, approximately 50 genes have been implicated in PD pathogenesis, including both Mendelian genes with rare mutations and low-penetrance genes with common polymorphisms. Previous studies of low-penetrance genes focused on protein-coding genes, and less attention was given to long noncoding RNAs (lncRNAs). In this study, we aimed to investigate the susceptibility roles of lncRNA gene polymorphisms in the development of PD. Therefore, polymorphisms (*n*=15) of the *PINK1-AS*, *UCHL1-AS*, *BCYRN1*, *SOX2-OT*, *ANRIL* and *HAR1A* lncRNAs genes were genotyped in Hungarian PD patients (*n*=160) and age- and sex-matched controls (*n*=167). The rare allele of the rs13388259 intergenic polymorphism, located downstream of the *BCYRN1* gene, was significantly more frequent among PD patients than control individuals (OR = 2.31; *p*=0.0015). In silico prediction suggested that this polymorphism is located in a noncoding region close to the binding site of the transcription factor HNF4A, which is a central regulatory hub gene that has been shown to be upregulated in the peripheral blood of PD patients. The rs13388259 polymorphism may interfere with the binding affinity of transcription factor HNF4A, potentially resulting in abnormal expression of target genes, such as *BCYRN1*.

## 1. Introduction

Parkinson's disease (PD) is the second most common neurodegenerative disease, which belongs to the group of motor system disorders. The key pathological hallmark of PD is the progressive loss of dopamine-producing cells in the substantia nigra pars compacta (located in the midbrain), which results in a dopamine depletion in the striatum. This biochemical imbalance manifests with cardinal motor symptoms including resting tremor, muscle rigidity, bradykinesia, and postural instability [1]. PD affects all populations worldwide with the prevalence of 1-2% among individuals over 65 years of age [2, 3]. Despite the numerous attempts of the last decades, there is still no known cure for the disease [4].

PD is a genetically heterozygous disease. A small portion of PD cases is familial, which can be caused by highly penetrant mutations in Mendelian genes, both with autosomal dominant (*SNCA*, *LRRK2*, *VPS35*, *EIF4G1*, and *CHCHD2*) and with autosomal recessive (*parkin*, *PINK1*, *DJ1*, *ATP13A2*, *FBXO7*, *PLA2G6*, and *DNAJC*) modes of inheritance [5, 6]. Mutations in some of these genes may also play a role in cases that appear to be sporadic. However, all known monogenic forms of PD explain only about 30% of familial and 3–5% of sporadic cases [7].

The sporadic forms of PD are linked to polymorphisms of low-penetrance genes [6] identified by case-control studies and lately in genome-wide association studies (GWAS). The single-nucleotide polymorphisms (SNPs) of these susceptibility loci contribute to the development of polygenic PD forms and are referred as risk variants for the disease. The polymorphisms located within common susceptibility variants—the *SNCA*, *MAPT*, *LRRK2*, *GBA*, *PARK16*, *BST1*, *DGKQ*, and *STK39* genes—exhibit the strongest association with sporadic PD [8–11]. These previous studies focused on polymorphisms of protein-coding genes, and little attention was given to polymorphisms of noncoding genes.

Long noncoding RNAs (lncRNAs), defined as nonprotein-coding RNA transcripts longer than 200 nucleotides, are emerging as key regulators of diverse cellular processes [12]. Certain lncRNAs are abundantly expressed in the cells of the central nervous system, providing an additional regulatory layer for fine tuning the cellular outcomes necessary for proper neuronal development and function [13]. Evidence is accumulating that lncRNAs have a pivotal role in PD development [14].

The antisense lncRNA of *PINK1* (*PINK1-AS*) is able to stabilize the expression of a *PINK1* splice variant in neurons and is involved in mitochondrial biogenesis [15]. The antisense lncRNA of the *UCHL* gene (*UCHL1-AS*) is under the regulation of Nurr1, which is a major transcription factor involved in dopaminergic cell differentiation and maintenance [16]. The *brain cytoplasmic RNA 1* lncRNA (*BCYRN1*, also referred to as *BC200*) gene is predominantly expressed in neural tissues and shows an elevated level in variety of tumor types [17] and in Alzheimer's disease (AD) brain samples [18]. BC200 lncRNA functions as translation repressor [19]. Another lncRNA implicated in PD is the overlapping transcript (*SOX2-OT*) of the *SRY-related HMG-box-2* gene (*SOX2*) which regulates the cotranscribed *SOX2* gene expression in neurogenesis and serves as a biomarker for neurodegeneration [14, 20]. The *highly accelerated region 1a (HAR1A)* gene marks the evolutionary divergence of humans and chimpanzees and plays a pivotal role in cortical-neuron specification and migration [21]. *ANRIL*, the antisense lncRNA of the *cyclin-dependent kinase inhibitor 2b (CDKN2B)* gene, is involved in the development of the melanoma and neural system tumor syndrome, familial melanoma syndrome, and various tumor types; however, its association with PD has not yet been investigated [22].

Although reported data suggest that lncRNAs regulate gene expression in the central nervous system, little is known about the role of their common gene variants in the pathogenesis of PD. Therefore, we aimed to investigate whether polymorphisms located in or close to the *PINK1-AS*, *UCHL1-AS*, *BCYRN1*, *SOX2-OT*, *ANRIL*, and *HAR1A* lncRNA genes are associated with PD.

## 2. Patients and Methods

### 2.1. Investigated Individuals

The patients (*n*=160) participating in this study were recruited from the Department of Neurology, University of Szeged, Szeged, Hungary, and from NEPSY Biobank of the Institute of Genomic Medicine and Rare Disorders at Semmelweis University, Budapest, Hungary. All patients fulfilled the diagnostic criteria for PD. Patients and age- and sex-matched healthy controls (*n*=167) were of Hungarian ancestry. The average age was 66.4 ± 9.31 years for PD patients and 64.9 ± 8.46 years for healthy controls. The percentage of males was 32.53% for PD patients and 45.50% for controls. The investigation was approved by the Internal Ethical Review Board of the University of Szeged and the Ethical Committee of Semmelweis University. Written informed consent was obtained from patients and healthy controls, and the study was conducted according to the Principles of the Declaration of Helsinki.

### 2.2. Genotyping

Four lncRNA genes previously implicated in PD *(PINK1-AS (NR_046507)*, *UCHL1-AS (KR709885)*, *BCYRN1 (NR_001568)*, and *SOX2-OT (NR_075091))* and two lncRNA genes linked with neuron differentiation and migration *(ANRIL (AB548314)* and *HAR1A (NR_003244))* were chosen for genotyping. Polymorphisms selected for genotypic analysis had not been previously studied in any neurodegenerative disorders and were present at a high frequency (global minor allele frequency >0.1) in European populations. The following 15 lncRNA variations were selected for analysis: *PINK1-AS* polymorphisms rs542589, rs1043424, and rs540038; *HAR1A* SNPs rs6089838, rs750697, and rs750696; *ANRIL* polymorphisms rs10738605 and rs564398; *BCYRN1* SNPs rs10865224 and rs13388259; *SOX2-OT* polymorphisms rs6765739 and rs13096623; and *UCHL1* SNPs rs12649180, rs17443616, and rs2342526.

Genomic DNA was isolated from peripheral blood samples using the DNeasy Blood and Tissue Kit (QIAGEN; Hilden, Germany). Genotyping of the lncRNA polymorphisms was based on allelic discrimination assays using the TaqMan SNP Genotyping Assay following the manufacturer's instructions (Thermo Fisher Scientific; Rockford, USA). Each genotyping plate contained samples genotyped in duplicate across all plates. Except *HAR1A* rs750696, all SNPs passed quality control criteria (sample call rate 80%, SNP efficiency > 95%, SNP genotyping accuracy > 99.5%).

### 2.3. Statistical Analysis

Statistical analysis of PD patients and controls was carried out according to the guidelines of case-control allelic association study design. The statistical significance of the association between the examined SNPs of the investigated lncRNA genes and PD was determined with the Fisher exact probability test. The Bonferroni correction for the multiple hypothesis of the analyzed SNPs (*n*=14) was also defined. For all SNPs, odds ratios (OR) with 95% confidence intervals (CI) were also determined. All statistical analyses were performed with VassarStats (http://www.faculty.vassar.edu/lowry/VassarStats.html).

## 3. Results

The 14 polymorphisms passing the quality control criteria were initially assessed in 101 PD patients and 83 controls enrolled at the Department of Neurology and Department of Dermatology and Allergology, University of Szeged, Szeged, Hungary. Three polymorphisms showed promising results with respect to allele distribution among PD patients and controls (Supplementary Table 1). The strongest association with PD was observed for the *BCYRN1* rs13388259 polymorphism (OR = 4.43, CI = 2.0–9.8, Fisher exact probability test *p*=0.00004), and notable results were observed for the *SOX2-OT* rs6765739 SNP (OR = 1.38, CI = 0.9–2.1, Fisher exact probability test *p*=0.0791) and the *UCHL1* rs12649180 SNP (OR = 1.63, CI = 0.9–3.0, Fisher exact probability test *p*=0.0907). No other investigated SNPs exhibited association with PD with respect to allele distribution in the two groups.

**Figure 1:**
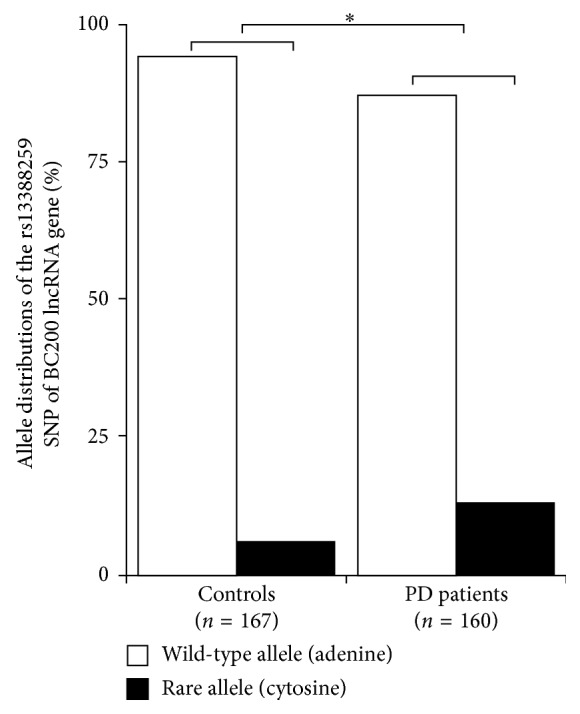
Allele distribution of the of the rs13388259 SNP of the *BC200* lncRNA gene among PD patients (*n*=160) and controls (*n*=167).

Due to these encouraging initial results, further PD patients and control individuals were enrolled into the study at the Department of Neurology, University of Szeged, as well as at the Institute of Genomic Medicine and Rare Disorders, Semmelweis University, Budapest, Hungary. The *BCYRN1* rs13388259 SNP, the *SOX2-OT* rs6765739 SNP, and the *UCHL1* rs12649180 SNP were assessed. For this extended study group (*n*=327; 160 PD patients and 167 healthy controls), the *BCYRN1* rs13388259 SNP again showed strong association with PD, and its rare allele was significantly more common among PD patients than control individuals (OR = 2.31, CI = 1.3–4.0, Fisher exact probability test *p*=0.0015, Bonferroni correction *p*=0.021) (Figure 1). The *SOX2-OT* rs6765739 SNP also showed notable, but not significant differences in allele distribution between PD patients and controls (OR = 1.28, CI = 0.9–1.8, Fisher exact probability test *p*=0.0666). The alleles of the *UCHL1* rs12649180 SNP did not exhibit notable differences in the distribution in PD patients and controls (Table 1).

The majority of SNPs are located in noncoding regions including introns or intergenic regions [23]. The disease association of SNPs located in noncoding regions sometimes is difficult to interpret. Using different sources, multiple in silico analyses were performed to gain information about the putative functional consequences of the SNP.

According to the Ensembl (http://www.ensembl.org/index.html), UCSC Genome Browser/SNP 147 (http://www.genome.ucsc.edu/), VarSome (https://www.varsome.com), and NCBI/refSNP (https://www.ncbi.nlm.nih.gov/SNP/) databases, rs13388259 has been assigned the functional class of intron variant within *BCYRN1* (ENSG00000236824.1), whereas ALFRED (https://www.alfred.med.yale.edu/alfred/index.asp) and F-SNP (http://www.compbio.cs.queensu.ca/F-SNP) database refer to rs13388259 as an intergenic SNP between the *BCYRN1* and *EPCAM* genes on Chromosome 2. According to the NCBI/SNP Database, the rs13388259 polymorphism is located on chr2:47,343,700 (GRCh38). The difference between the predicted functions of the rs13388259 polymorphism is probably the consequence of the miss-annotation of the *BCYRN1* gene and transcript in various databases. By comparing the data in several databases, we found that two overlapping genes and their corresponding transcripts have been given the name BCYRN1 (BC200): a gene 13,458 nt in length (chr2:47,331,060-47,344,517, GRCh38, Gencode Transcript ENST00000418539.1, and Gencode Gene ENSG00000236824.1) and a gene 200 nt in length (chr2:47,335,315-47,335,514, GRCh38, Gencode Transcript ENST00000418539.2, and Gencode Gene ENSG00000236824); the two genes are in the same orientation.

To evaluate if the region containing the rs13388259 polymorphism is transcribed, we designed a primer pair for real-time PCR of that region. Using total RNA isolated from the peripheral blood of healthy individuals (*n*=3) and chronic lymphoid leukemia (CLL) patients (*n*=3), cDNA reverse transcription was performed followed by a real-time PCR using the designed primers: no PCR product was detected for the region containing the SNP, although a control using primers for the GAPDH gene was successful (data not shown). Using a primer pair designed for the 200 nt transcript, a PCR product was obtained in samples derived from CLL patients but not in samples from healthy controls (data not shown). This result is in agreement with previous findings that, although BC200 is expressed in normal neural tissue, it is expressed at higher levels in various tumor types. Based on this RT-PCR result, we suppose that rs13388259 is located in an intergenic region.

**Figure 2:**
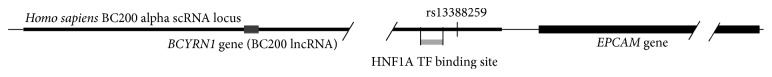
The genomic region on Chromosome 2 in which rs13388259 occurs. The rs13388259 SNP is an intergenic polymorphism located on the short arm of Chromosome 2 (Ch2:47,343,700) between the *BCYRN1* and *EPCAM* genes. The 200 nt *BCYRN1* lncRNA gene is located at positions 47,335,315-47,335,514 (ENSG00000236824) and overlaps with the *Homo sapiens* BC200 alpha scRNA locus (accession number AF020057.2; 13,472 bp length; position 47,331,060-47,344,531). The *EPCAM* gene (Chr2:47,345,158-47,387,601; ENSG00000119888) is located 1458 bp downstream of the SNP. The HNF4A transcription factor binding site is located approximately 236 bases upstream of the rs13388259 SNP (Ch2:47,343,088-47,343,464). Genomic positions are relative to GRCh38.

Results from analysis with the F-SNP software indicated that the rs13388259 SNP is associated with transcriptional regulation. TFBIND software (http://www.tfbind.hgc.jp/) was applied to predict transcription factor binding sites [24], and the analysis revealed that the single-nucleotide alteration in the rs13388259 region (A/C) possibly modifies the binding affinity of a putative transcription factor binding site. To determine the relationships between the identified SNP and regulatory sequences, annotations of regulatory elements containing binding sites were downloaded from the UCSC genome browser [25]. The rs13388259 SNP is located 236 bp downstream of a binding site for the hepatocyte nuclear factor 4 alpha (HNF4A) transcription factor. According to the ORegAnno DNA regulatory region database, this binding site might be functionally related to the *BCYRN1* gene (OREG1716976 and OREG1741230) (Figure 2). Furthermore, an enhancer region (ID GH02H047342, localization chr2:47,342,469-47,347,454) described in the GeneCard Human Gene Database (http://www.genecards.org) that regulates both the *BCYRN1* and the *EPCAM* genes is located near the rs13388259 SNP. This enhancer region contains binding sites for transcription factors, including HNF4A. The HNF4A transcription factor is associated with gluconeogenesis and diabetes and has been identified as a central regulatory hub gene upregulated in the peripheral blood of PD patients [26].

## 4. Discussion

As human life spans are prolonged, the incidence of neurodegenerative diseases is increasing. PD is the second most common neurodegenerative diseases worldwide (after Alzheimer's disease) and is currently the most important known risk factor of the elderly with only symptomatic treatment [2, 3]. Therefore, insight into the mechanism of PD is essential. In this study, we contribute to the understanding of the putative roles of lncRNAs, which provide an additional level of gene expression regulation in the development of sporadic, polygenic PD.

We compared the distribution of the rare and wild-type alleles of 15 polymorphisms of the *PINK1-AS*, *UCHL1-AS*, *BCYRN1*, *SOX2-OT*, *ANRIL*, and *HAR1A* lncRNA genes in Hungarian PD patients and ethnicity-, age-, and sex-matched controls. Our results demonstrated strong association between the presences of the rs13388259 intergenic polymorphism and PD. This intergenic SNP is located between the *BCYRN1* and *EPCAM* genes on Chromosome 2, and a functional link to *BCYRN1* has been annotated for the region (http://www.varsome.com, http://www.noncode.org).

The *BCYRN1* lncRNA gene arose after the separation of monkeys and humans in the mammalian linage as a consequence of the recruitment of the monomeric *Alu* element, which was subjected to retro transposition from an active master gene 35–55 million years ago [27, 28]. BC200 is primate tissue-specific RNA polymerase III transcript [29] exhibiting brain-specific expression in transgenic mice [30]. The BC200 lncRNA is involved in local translational control [31, 32]. Dysfunction of the BC200 lncRNA in neurons—due to either altered expression or mislocalization—results in the deregulation of dendritic mRNA expression, failure of long-term synaptic plasticity, and thus, neurodegeneration [14, 18, 33].

The BCYRN1 lncRNA listed as a 200 nt transcript in the Ensemble Genome Browser (ID: ENST00000418539.2, http://www.ensemble.org) [34]. The rs13388259 SNP is located within an untranscribed region downstream of the BC200 lncRNA and replaces adenine with cytosine (NG_012352.2:g.3538A>C). A binding site for the HNF4A transcription factor is located 236 nt upstream of the SNP, and a short interspersed nuclear element (AluSx) is located 73 nt downstream.

The minor allele frequency (MAF) for the cytosine variant is 0.0865 in the European and in the American population and 0.1807 in Africans. No known clinical significance of this polymorphism has previously been identified by GWAS studies of PD or any other disease.

Previous studies have confirmed that genetic variants in the primary sequence of the lncRNA genes (noncoding RNA transcripts) are highly correlated with human diseases [35, 36]. However, it is difficult to determine the contribution of intronic and intergenic SNPs to the development of disease since these are located outside of the primary RNA sequences. The expression pattern of many lncRNAs shows spatial and temporal specificity, pointing to the strong regulation of lncRNAs expression [37]. Abnormal expression of several lncRNAs is linked to human diseases, for example, elevated level of particular lncRNAs expression closely correlates with several types of cancer formation and/or metastatic activity [38, 39]. SNPs of noncoding genomic regions—either intronic or intergenic—are often located in or closely linked to regulatory regions [23]. They may interfere with host regulatory elements [40, 41] and may affect the expression level of lncRNAs implicated in the pathogenesis of certain diseases. The results of our in silico analysis demonstrated that the *BCYRN1* rs13388259 polymorphism lies close to the binding site of the transcription factor HNF4A. HNF4A was identified as a central regulator hub gene upregulated in peripheral blood of PD patient [42]; moreover, the relative abundance of the HNF4A mRNA correlates with the severity of the disease: upon 3-year follow-up constantly increasing HNF4A expression was observed [26]. The *BCYRN1* was predicted as a target gene of HNF4A transcription factor binding site. These data together suggest that the rs13388259 polymorphism may modify the expression level of BC200 lncRNA due to the modification of HNF4A transcription factor binding affinity.

Previous studies linked BC200 lncRNA with neurodegeneration [18, 36]. This is the first study, however, which confirms the genetic association between the genomic context of the *BCYRN1* lncRNA gene and PD. Our study further emphasizes the increasing awareness of the significance of lncRNAs in the development of human diseases. Further studies are needed to confirm the functional relevance of the identified genetic variants in the expression and/or activity of the BC200 lncRNA and its functions in dopaminergic neurons.

## Figures and Tables

**Table 1:** Genotype and allele distributions of the rs13388259 SNP of the *BC200* lncRNA gene, the rs6765739 SNP of the *SOX2-OT* lncRNA gene, and the rs12649180 SNP of the *UCHL1* lncRNA gene in PD patients (*n*=160) and controls (*n*=167).

	Genotypes (*n*)			Alleles (*n*)		Statistical analysis		
	Homozygous wild type	Heterozygous	Homozygous rare	Wild type	Rare	Odds ratio	95% confidence interval	Fisher exact probability test
rs13388259 SNP of the *BC200* lncRNA gene								
PD patients (*n*=160)	117	43	0	277	43	**2.31**	1.3–4.0	*p*⁡**=0** **.0015**
Controls (*n*=167)	147	19	1	313	21			
rs6765739 SNP of the *SOX2-OT* lncRNA gene								
PD patients (*n*=160)	17	139	4	173	147	1.28	0.9–1.8	*p*=0.0666
Controls (*n*=167)	35	131	1	201	133			
rs12649180 SNP of the *UCHL1* lncRNA gene								
PD patients (*n*=160)	126	34	0	287	34	1.21	0.8–2.0	*p*=0.2516
Controls (*n*=167)	125	42	0	292	42			

## Abbreviations

ANRIL: Antisense lncRNA of the CDKN2B
BC200/BCYRN1: Brain cytoplasmic RNA 1 lncRNA
CDKN2B: Cyclin-dependent kinase inhibitor 2b
HAR1A: Highly accelerated region 1a
lncRNAs: Long noncoding RNAs
PD: Parkinson's disease
PINK1: Phosphatase- and tensin homologue-induced putative kinase 1
PINK1-AS: Antisense lncRNA of PINK1
SNCA: *α*-synuclein
SNP: Single nucleotide polymorphisms
SOX2: SRY-related HMG-box 2 gene
SOX2-OT: Overlapping transcript of the SOX2
UCHL1: Ubiquitin carboxy-terminal hydrolase L1
UCHL1-AS: Antisense lncRNA of the UCHL1.

## Supplementary Materials

Supplementary Materials Table 1: allele distribution of the 14 investigated polymorphisms in PD patients (*n*=101) and controls (*n*=83).
