# Supplementary material for: The rs13388259 Intergenic Polymorphism in the Genomic Context of the BCYRN1 Gene Is Associated with Parkinson's Disease in the Hungarian Population
Source: Parkinsons Dis. 2018 Apr 3;2018:9351598. doi: 10.1155/2018/9351598 (PMC5903343; doi:10.1155/2018/9351598)
Supplement: Supplementary Materials — Table 1: allele distribution of the 14 investigated polymorphisms in PD patients (n=101) and controls (n=83). [file 9351598.f1.pdf]

Supplementary Table 1. Allele distribution of the 14 investigated polymorphisms in PD patients (n=101) and controls (n=83).

		PD patients (n=101)		Controls (n=83)		Statistical analysis		
**LncRNA**	**SNP**	Wild type allele (n)	Rare allele (n)	Wild type allele (n)	Rare allele (n)	**Odds ratio**	**95% Confidence interval**	**Fisher exact probability test**
PINK1-AS	rs542589	181	21	147	19	1.11	0.6–2.2	p=0.4373
	rs1043424	156	46	133	33	1.19	0.7–2.0	p=0.2935
	rs540038	182	20	146	20	1.25	0.6–2.4	p=0.3110
HAR1A	rs6089838	107	95	88	78	1.01	0.6–1.5	p=0.5387
	rs750697	153	49	117	49	1.31	0.8–2.1	p=0.1544
ANRIL	rs10738605	126	76	106	60	1.07	0.7–1.6	p=0.4274
	rs564398	119	83	92	74	1.15	0.8–1.7	p=0.2851
BC200	rs10865224	119	83	97	69	1.02	0.7–1.5	p=0.5053
	**rs13388259**	**165**	**37**	**158**	**8**	**4.43**	**2.0–9.8**	**p=0.00004**
SOX2-OT	rs13096623	183	19	150	16	1.03	0.5–2.1	p=0.5390
	**rs6765739**	**107**	**95**	**101**	**65**	**1.38**	**0.9–2.1**	**p=0.0791**
UCHL1	**rs12649180**	**183**	**19**	**142**	**24**	**1.63**	**0.9–3.0**	**p=0.0907**
	rs17443616	144	58	126	40	1.27	0.8v2.0	p=0.1899
	rs2342526	129	73	114	52	1.24	0.8–1.9	p=0.1951
